# Clinic Atlanta Medical College

**Published:** 1874-05

**Authors:** J. G. Westmoreland, J. G. McKinney

**Affiliations:** Prof. of Materia Medica and Therapeutics; Reporter


### Clinic Atlanta Medical College.

---

### MEDICAL.

*J. G. Westmoreland, M.D., Prof. of Materia Medica and Therapeutics.*

J. G. McKINNEY, Reporter.

[*Continued from page 576, vol. xi, of the Journal.*]

November 24th, Emma Green, returned. Auscultation and percussion reveal the same difficulty of the right lung, with increased activity, amounting to puerile respiration of the left—more cough, less dulness.

This case, gentlemen, affords a full opportunity for practicing the ear in physical diagnosis. Here we have the vesicular murmur magnified, so as to impress the inexperienced very forcibly when the left side is examined, and a comparison of the two sides gives such marked difference that none will fail to perceive it.

You will have an opportunity to practice the physical signs of diagnosis in this case, after the lecture hour; and I desire that all participate in the examination. There are several methods of auscultation. *Mediate* is that in which a tubular instrument, called a stethescope, is placed between the ear of the investigator and the chest of the patient. The funnel shapped extremity is applied to the surface so as to press equally in its whole circumference, and collects the sound which is conveyed through the tube to the ear at the other extremity. The instrument is sometimes arranged with two flexible tubes proceeding from the cup or funnel-shaped extremity for insertion into both ears. A cylinder of wood is the simplest form of stethescope, and answers the purpose very well.

*Immediate* auscultation is, however, generally preferable, when the rules of propriety do not interfere with application of the ear directly to the chest. To one not accustomed to such examination, this mode gives the sounds much more distinctly, yet the exact point of any particular circumscribed local disturbance can be more accurately described by an instrument.

For percussion a pleximiter, consisting of a copper or other hard metalic plate, and a leaden hammer, are used. The plate, in order to give the proper sound, should press accurately upon the surface. The middle finger of the left hand, however, answers every purpose of a plate, and a finger of the other, flexed at right angle with the hand, serves for the hammer.

Resonance is natural when a healthy chest is struck, but dulness of sound by percussion indicates a more solid consistence of the contents.

Hence, in the case before us, as you observe, there is decidedly more resonance of the left side, because of the cellular structure of the lung, and its inflation with air. On the other side we find dulness from filling up of cells with supposed tuberculous substance, more dense than air.

In practicing auscultation of the respiration, on this subject, it will be perceived that a sound is heard in the diseased right side, differing in tone and intensity from that of the left, and evidently also in the distance of its origin, from the ear. This sound heard in the diseased side is produced by the air passing through the bronchia in making its way to the small portions of lung not solidified, and is called bronchial sound. That sound most distinctly heard in the left side is caused by the air entering the cells, and is called vescicular murmur.

The distinction between these two sounds may be practically studied in this case.

Auscultation has also to do with sounds of the voice. When the ear is applied to the right side, and the patient directed to talk, quite a different impression is made from that of the left The diseased side conveys to the ear the sensation produced by the mouth of the speaker placed near the ear. This is the result of a solid lung intervening between the bronchia, conveying the sound between the vocal apparatus and the ear, which conducts it more readily than healthy lungs, and at the same time there is no vesicular murmur to neutralize or destroy it.

This sound of the voice thus reaching the ear through the dense lung is called bronchophony. Another and very different condition leads to a sound somewhat similar, If, instead of solid lung, a cavity existed in the upper or central portion, from destruction of its substance, a sound would be made by the voice, for which bronchophony is liable to be mistaken. This is called pectoriloquy, and is distinguished from the former by percussion, it giving more resonant sound than should be expected from solid lung beneath.

In continuing the treatment of this case, we prescribe iodine by inhalation once a day. No expensive apparatus is necessary for this purpose; and while simple means are not always most attractive to patients, we should content ourselves with having effected, in the most economical manner, the greatest amount of benefit. As before described to you, we directed that an ordinary morphine vial, with wide mouth, be furnished, and when heated sufficiently, a grain or two of iodine is to be put in it and the fumes inhaled through the nostrils or mouth with sufficient force to reach the lungs.

We have two objects to accomplish by this procedure. The local catheretic effect of the remedy upon the diseased respiratory organs, and a catalytic influence that may probably be exerted upon the tuberculous deposit. Iodide potassium internally continued.

December 13th. No very decided change in the symptoms except that coughing is more frequent and expectoration freer. Inhalation of iodine continued.

December 25th. Sound of percussion on right side much more resonant; vesicular murmur quite perceptible; cough has ceased. General appearance greatly improved. Iodide potassium by the stomach, in the dose of five grains three times a day, directed.

January 12th. Dullness on percussion a little more decided, yet vesicular murmur quite distinct. General appearance still more improved. Prescribed iodine by inhalation every morning.

February 5th. No cough, looks very well, has good apetite and digests well, but still has dullness on percussion over apex of right lung and want of perfect respiratory murmur.

Directed to discontinue the treatment for two weeks.

